# Cognitive flexibility, impulsivity, and compulsivity in problematic consumption behaviours

**DOI:** 10.1371/journal.pone.0354596

**Published:** 2026-07-23

**Authors:** María Jara-Rizzo, Daniel Oleas, Jose A. Rodas

**Affiliations:** 1 Facultad de Ciencias Psicológicas, Universidad de Guayaquil, Guayaquil, Ecuador; 2 Dirección de Investigación, Universidad ECOTEC, Samborondón, Ecuador; 3 Department of Social Anthropology, Basic Psychology and Public Health, Pablo de Olavide University, Seville, Spain; 4 Escuela de Psicología, Universidad Espíritu Santo, Samborondón, Ecuador; 5 School of Psychology, University College Dublin, Dublin, Ireland; Kyoto University Graduate School of Informatics: Kyoto Daigaku Daigakuin Johogaku Kenkyuka, JAPAN

## Abstract

Different studies have identified cognitive inflexibility, impulsivity and compulsivity in substance use and gambling disorder, but it is unclear whether these deficits are present in other possible addictive behaviours. In this study, we evaluated cognitive inflexibility, as measured by the Probabilistic Reversal Learning Task (PRLT), impulsivity across UPPS-P model and the compulsivity in regular gamblers, buyers, video gamers, consumers of pornography and control group. Multivariate analysis was performed to evaluate the five groups, revealing higher scores across several impulsivity and compulsivity domains in the buyers group compared to the other groups. On the overall measure of cognitive flexibility using the PRLT, buyers performed worse than gamers and pornography users, suggesting a relative disadvantage in adapting to changing reward contingencies even before trial-level learning patterns were examined. To further evaluate patterns of cognitive flexibility across groups, a GLME model was developed. The results showed that phase-group interactions were generally weak, and none of the contrasts related to reversal differed between groups. This indicates that the cost of adapting to contingency reversals was similar across all five groups, with no evidence that any particular group would exhibit greater inflexibility during reversal phases. Finally, the findings show that impulsivity and compulsivity can manifest themselves differently in these activities, which has implications for the development of more personalised intervention strategies that take into account both the nature of the disorder and the specific cognitive deficits involved.

## Introduction

In the last two decades, a notable surge in scientific interest has been observed regarding the study of problematic behaviours associated with the excessive engagement in highly rewarding activities [1], such as gambling, video gaming, online pornography, and compulsive buying [2]. While these behaviours are common in the general population, a subgroup of individuals manifests patterns characterised by loss of control, a progressive increase in involvement, compulsive tendencies, and difficulty discontinuing the behaviour despite experiencing negative consequences [3]. These patterns often interfere—even if mildly or moderately—with emotional well-being [4], academic [5] or occupational performance [6], and social relationships [7]. Furthermore, under certain conditions where functional impairment is significant, they are considered non-substance addictions or behavioural addictions [8]. Consequently, problematic consumption behaviours have become a growing public health concern and a priority area of interdisciplinary research, even though the majority lack formal diagnostic recognition. Their increasing prevalence and potential adverse impact underscore the need to rigorously investigate the psychological and cognitive mechanisms that underlie their development.

From a nosological perspective, the diagnostic status of these behaviours is heterogeneous and continues to be a subject of debate. Firstly, there is at least one consensus: Gambling Disorder is recognized as an addictive disorder in both the DSM-5-TR [9] (Substance-Related and Addictive Disorders) and the ICD-11 [10] (Disorders due to addictive behaviours). Secondly, the case of video gaming illustrates the lack of convergence between classification systems: while the ICD-11 incorporated Gaming Disorder as a disorder due to addictive behaviours, the DSM-5-TR maintains Internet Gaming Disorder in Section III as a condition requiring further research before full diagnostic inclusion. Outside of these entities, other behaviours of interest present an even more uncertain status. Compulsive buying/shopping lacks an independent diagnostic category in the DSM-5 and, in the ICD-11, it is considered a phenomenon that can be placed—where appropriate—within residual categories of the impulse control disorders spectrum, rather than within addictive disorders. Similarly, the DSM-5 does not recognise a specific diagnosis for problematic pornography use; its discussion is usually linked to broader constructs of sexual compulsivity. In the ICD-11, Compulsive Sexual Behaviour Disorder is included within impulse control disorders, reflecting a cautious stance regarding its classification as an addiction.

This classification landscape sustains a central debate: whether these forms of excessive consumption represent genuine expressions of behavioural addiction or if, conversely, there is a risk of over-pathologising everyday activities when diagnostic labels are broadened without strict criteria for severity and functional impairment [11] (e.g., Argentine tango [12]). Consequently, this diagnostic uncertainty reinforces the relevance of investigating underlying mechanisms to differentiate between high normative involvement and patterns consistent with an addictive architecture. Grouping these behaviours, despite their varied diagnostic statuses, enables a transdiagnostic evaluation. This approach facilitates the identification of shared cognitive mechanisms, independent of current nosological boundaries, to determine if a common addictive architecture underlies disparate consumption patterns.

In the study of addictions, it has been proposed that certain psychological and neurocognitive processes play a central and relatively stable role across distinct addictive expressions [13]. Among these processes, impulsivity, compulsivity, and cognitive flexibility stand out. These are conceived as domains with potential transdiagnostic value for explaining both the initial vulnerability and the persistence of the behaviour [14].

Within this framework, impulsivity is defined as the tendency to respond prematurely or in an insufficiently planned manner, characterised by a preference for immediate gratification and difficulties in inhibitory control, which can translate into short-term oriented decisions and diminished behavioural regulation [15]. Compulsivity, in turn, refers to the rigid persistence of repetitive behaviours that are maintained despite negative consequences, featuring a marked difficulty in resisting or interrupting them and a progressive automatisation of the behavioural pattern [16]. Finally, cognitive flexibility refers to the capacity to adjust strategies, change criteria or action rules, and abandon habits when they cease to be functional; its impairment is associated with rigidity, perseveration, and difficulties in learning from feedback, which can favour the continuity of maladaptive behaviours [17]. Composed, these three processes offer an integrative framework for characterising the shared cognitive architecture of addictions, as they simultaneously capture the tendency toward reward-oriented discontrol (impulsivity), rigid persistence despite harm (compulsivity), and the difficulty in readjusting behaviours in response to contextual changes (inflexibility).

Regarding Gambling Disorder (gambling), meta-analyses demonstrate a consistent profile across all three domains: increased impulsivity [18], compulsivity-related traits/alterations, and diminished cognitive flexibility when compared with controls [19]. In gaming disorder, profiles have been described where impulsive and compulsive components coexist [20], as well as signs compatible with compulsive traits and diminished flexibility [21]. In problematic pornography use, findings are primarily concentrated on impulsivity and sensation seeking [22]. However, the available empirical basis for Problematic Sexual behaviours is generally heterogeneous and limited, with evidence regarding compulsivity as a differentiated domain often ranging from weak to moderate, the construct of cognitive flexibility has yet to be systematically investigated in this population, thus precluding firm conclusions [23,24]. Finally, compulsive buying/shopping is consistently reported to feature a strong impulsive component and conceptual overlaps with both compulsive and “addiction-like” traits [25]; in contrast, cognitive flexibility, has been less explored, and the associated deficits, when observed, tend to be context-dependent, primarily emerging in the presence of buying cues (or shopping-related stimuli). Collectively, these findings establish associative relationships between the constructs, but they fail to provide a systematic comparison either between the distinct problematic behaviours or the underlying neuropsychological processes.

At a comparative level among behavioural addictions, the available evidence allows for the delineation of relative patterns for impulsivity and compulsivity, and—with greater caution—for cognitive flexibility. Regarding impulsivity, a broad clinical comparison across various behavioural addiction phenotypes suggests that novelty seeking, as a trait closely linked to impulsivity, tends to be highest in compulsive buying, intermediate in gambling and compulsive sexual behaviours (like pornography users), and lowest in gaming [26]. Nevertheless, this hierarchy is not entirely stable: at least one specific comparative study reports an inverse pattern between gaming and gambling in clinical samples [27]. With respect to compulsivity, comparative evidence based on related indicators suggests a pattern in which compulsive sexual behaviours and compulsive buying tend to concentrate the highest levels [28], while gambling and gaming show relatively lower levels or a less pronounced profile [26]. Regarding cognitive flexibility, direct comparability is even more limited. A study with a transdiagnostic approach suggests that Gambling Disorder presents a small deficit in flexibility, whereas compulsive buying and compulsive sexual behaviour do not emerge as clearly comparable deficit-driven profiles under that framework [29]. For gaming, other evidence suggests more marked inflexibility than in gambling and, in relative terms, higher than that observed in buying or sexual behaviour. However, this ordering must be interpreted with caution, as it stems from distinct studies with non-equivalent operationalisations and metrics [21].

In summary, although it is possible to propose preliminary comparative trends, the available literature does not yet allow for the establishment of a definitive gradient across behaviours when methodological equivalence in the measurement of impulsivity, compulsivity, and cognitive flexibility is required.

Collectively, the available evidence suggests that impulsivity, compulsivity, and cognitive flexibility are relevant dimensions across various behavioural addictions; however, current knowledge remains fragmented for at least three reasons. First, the majority of studies have been conducted per behaviour, describing profiles within gaming, gambling, pornography/CSB, or buying, rather than establishing direct comparisons between them. Second, where comparisons do exist, they tend to rely on proxy indicators (e.g., personality traits or associated symptoms) or non-equivalent operationalisations across studies, which limits the interpretation of differences as true variations in neuropsychological mechanisms. Third, the focus on clinical or high-severity samples leaves it relatively unclear whether these processes also differentiate profiles at subclinical levels, where functional impairment may be mild or incipient, but the behavioural pattern already signals a loss of function as the foundation of an addictive neuropsychological architecture. In this context, the objective of the present study is to identify and compare differences in impulsivity, compulsivity, and cognitive flexibility among participants exhibiting subclinical addictive behaviours across various forms of problematic engagement, in order to evaluate the extent to which they share—or diverge in—the core processes proposed as part of an addictive architecture.

## Materials and methods

### Participants and procedure

A total of 630 participants were assessed between 17/may/2024 and 11/july/2025, distributed as follows: 78 buyers, 139 video gamers, 115 gamblers and 147 consumers of pornography and 151 adults who did not engage in any of the studied behaviours regularly. Initial recruitment was conducted via institutional digital noticeboards across four universities (University of Guayaquil, Central University of Ecuador, Ibero-American University of Ecuador, and ECOTEC University) situated in Ecuador’s two largest cities. Subsequent recruitment utilised a snowball sampling approach, wherein initial respondents distributed the study link within their broader social networks. Consequently, while the sample extends beyond university students to include general community members, it remains a predominantly young and educated cohort. All participants were assessed virtually through the PsyToolkit platform [30]. Before receiving the assessment link, potential participants were asked about inclusion criteria. The participants were then evaluated virtually and anonymously. After being informed about the evaluation process, they gave their consent by clicking o a button displaying “I agree to participate.”

The inclusion criteria for all groups were being aged 18 years or older, engaging regularly in the respective behaviours (at least twice a month for the past 12 consecutive months), and presenting a subclinical status. Subclinical status was operationalised through the community-based nature of the sample, the absence of previous or current treatment for addiction-related disorders, the lack of formal diagnosis of neurodevelopmental, neurocognitive, or addictive disorders, and the self-reported preservation of daily functioning. Consequently, participants exceeding psychometric risk cut-offs were retained, as these thresholds denote high-risk engagement rather than confirmed clinical pathology in the absence of a clinical interview and evident functional impairment.

### Ethical approval and informed consent statements

This study is part of the FCI-058–2023 Project, approved by the University of Guayaquil, Ecuador. University High Council resolution (No. R-CSU-UG-SE34-313-14-09-2023) on Month 09, 2023. Additionally, the project has received ethical and scientific approval from the Research Committee of the Psychology Programme at Universidad Ecotec, Ecuador, CERTIFICATE No. 16-08-03-2024. The study complies with ethical standards and research protocols in psychology, ensuring participant protection in accordance with the Declaration of Helsinki of 1964 and its subsequent amendments.

### Instruments

All participants were evaluated with the following questionnaires:

*Ad-hoc initial questionnaire*: this instrument was developed to collect demographic information (age, sex, household net income, and highest level of education completed). It also included selection questions based on the inclusion criteria for the 5 groups (control group, buying, video games, gambling, and pornography groups).

*Impulsive Behaviour Scale – UPPS-P*: the present study used a Spanish version that has shown good psychometric properties in previous studies [31], including Ecuadorian population [32], with Cronbach’s alpha ranging from .61 to .81, indicating acceptable internal consistency. Within the present sample, the overall scale demonstrated good internal consistency (α = .829). This questionnaire assesses impulsivity through 20 Likert-scale items. According to Whiteside and Lynam’s model, the five dimensions of impulsivity are: negative urgency, positive urgency, sensation seeking, lack of premeditation, and lack of perseverance.

*Compulsivity Assesment Questionnaire – GRACC18*: To assess compulsivity in this study we used the 18-item version of The Granada Assessment for Cross-domain Compulsivity questionnaire. This Likert-scale questionnaire was designed to evaluate compulsivity, particularly in relation to candidate behavioural addictions [33] and has been used in Ecuadorian population before [34]. The original study has shown excellent internal consistency, with a Cronbach’s alpha of .98. In the current sample, the instrument exhibited excellent internal consistency (α = .959).

*Probabilistic reversal learning task – PRLT*: To evaluate cognitive inflexibility we used the PRLT [35]. In the task, participants are presented with a choice in each trial between two differently coloured squares—green and blue—displayed simultaneously on a black screen. On each trial, participants must choose a coloured square with a click. The task is organised into four distinct phases, each consisting of 40 trials. During each phase, one coloured square is arbitrarily deemed the “correct” choice. Selection of the correct square results in the award of symbolic points 80% or 70% of the time, while selecting the “wrong” square leads to the deduction of points. In the first and third phases, the green square is designated as the correct choice 80% and 70% of the time, respectively. In contrast, the blue square is the correct choice in the second and fourth phases. This version has been successfully used as a cognitive flexibility measure in others studies in Ecuadorian population [36,37].

Prior to the analyses, all continuous variables were standardised. Extreme univariate outliers, defined as scores exceeding ±3 standard deviations from the mean, were subsequently removed from the dataset. This procedure was implemented to prevent extreme values from disproportionately influencing the multivariate estimates and to satisfy the distributional assumptions of the general linear model. While this trimming approach enhances statistical robustness, it is acknowledged that it may marginally attenuate natural clinical variance, potentially restricting the generalisability of the findings to the most extreme presentations of these behaviours.

To measure the severity of consumption based on each group, we used the following questionnaires:

*Diagnostic Questionnaire for Gambling Disorder - GD9*: This questionnaire was originally developed by Stinchfield [38] based on the DSM-IV-TR diagnostic criteria for Gambling Disorder (GD). It comprised 19 dichotomous items (yes = 1 point; no = 0 points), with two items assessing each of the ten diagnostic criteria, except for criterion 4, which was assessed by a single item. A total score of 4 or more was used as the diagnostic cut-off for GD. Within our sample, the scale demonstrated excellent internal consistency (α = .926).

*Diagnostic Questionnaire for Internet Gaming Disorder - IGD9*: The IGD9 is a 9-item scale evaluating internet gaming disorder symptoms, with its items representing each the 9 symptoms listed in the DSM-5-TR [9] for the disorder. In the original version of the scale [39] participants responded each item using a Likert-type response style. For the present study we used the dichotomous response version used by Muela et. al. [33] which has shown good internal consistency (α = .85). Within the current sample, the dichotomous version maintained acceptable internal consistency (α = .789). In this version, participants responded either “yes” (1) or “no” (0) to each of the nine items, yielding a total score ranging between 0 and 9. According to Muela et al., scores of 5 and higher are indicative of a possible Internet Gaming Disorder as proposed in Section III of the DSM-5-TR [9].

*Problematic Pornography Consumption Scale – PPCS*: The 18-item PPCS was developed based on the well-established six-component Griffiths model of addiction [40] to assess PPU: salience, Tolerance, Mood modification, Conflict, Withdrawal, Relapse. Beáta Bőthe et al. [41] performed a cross-cultural validation of the PPCS, which included an Ecuadorian sample. This questionnaire demonstrated excellent psychometric properties (α = 0.80–0.95). It is a seven-point scale (1 = never; 7 = all the time), with total scores ranging from 18 to 126 points. Scoring ≥ 76 points on the PPCS indicates being at risk of problematic pornography use. Within this study sample, the scale showed excellent internal consistency (α = .955).

*Pathological buying screener – PBS*: Fernández-Aranda et al. [42] evaluated the psychometric properties of the Spanish version of the PBS. It is a five-point scale (1 = never; 5 = very frequently) with 13 items distributed across 2 factors (excessive buying behaviour and loss of control) with an excellent Cronbach’s α (.92 and .86 respectively). Total scores range from 13 to 65 points; a score ≥ 39 on the PBS is an indicator of pathological buying risk. In the current sample, the total scale displayed excellent internal consistency (α = .954).

### Analysis plan

To characterise group differences in impulsivity, compulsivity and cognitive flexibility, and to examine trial-level learning dynamics in the PRLT a multivariate and mixed-effects analyses were planned. The first stage used a MANCOVA framework to assess whether the five behavioural groups differed on the combined set of psychological measures while adjusting for age and educational level. This multivariate approach was selected because the dependent variables were conceptually related and showed moderate intercorrelations, making it important to evaluate them jointly rather than through separate tests. Assumptions of linearity and multicollinearity were examined, and because the covariance matrices were not homogeneous across groups, Pillai’s trace was used as a robust test statistic. Significant multivariate effects were followed by univariate analyses and Holm-adjusted post hoc comparisons to determine the specific variables driving group differences and the pattern of contrasts among groups.

The second stage examined PRLT performance at the trial level using generalised linear mixed-effects models (GLME) with a binomial link as performed in Jara-Rizzo et al. [36]. This approach was chosen because PRLT accuracy is a binary outcome measured repeatedly within participants, and the design includes both within-participant factors (Phase, Trial) and a between-participant factor (group). Trial number was treated as a log-transformed continuous predictor to model learning as a curvilinear process and improve convergence. Phase was decomposed into three orthogonal contrasts to capture distinct components of task structure: differences between the first and second halves of the task (C1), the alternating effects of rule reversals between odd and even phases (C2), and the differences between the middle phases and the initial/final phases (C3). The initial saturated model included all main effects and their two- and three-way interactions, with participants modelled as a random factor and a random slope for Log-trial to capture individual differences in learning rate. A hierarchical model-comparison procedure based on likelihood ratio tests and AIC was then used to identify the most parsimonious fixed-effects structure supported by the data. Because the original random-effects specification produced a singular fit, alternative random structures were compared, and the final model retained the random slope for Log-trial, which reflected meaningful participant-level variability in learning.

## Results

The total sample consisted of 630 participants distributed into 5 groups. As shown in Table 1, the mean age of the buying, video game, and control groups is between 23 and 25 years, while the gambling group has a mean age of 32 years. Regarding the level of education completed, most participants in the 5 groups had completed secondary education. Participants were also asked about their monthly income, and most responded that they earned between $451 and $900. Finally, regarding the level of severity due to consumption, it was observed that 52.5% of the buying group obtained scores equal to or higher than the cut-off (≥ 39 points) for pathological buying, 52.9% of the gambling group reported scores equal to or higher than the cut-off (≥ 4 in) for gambling disorder, 14.3% of the videogame group evaluated obtained scores equal to or higher than the cut-off (≥ 5) for internet gaming disorder, and 11.2% of participants in the pornography group obtained scores equal to or higher than the cut-off point (≥ 76 points) for problematic pornography consumption.

**Table 1:** Demographic Variables and Severity Level due to Consumption.

	Buying group	Gambling group	Videogame group	Pornography group	Control group
Sex	20 men, 58 women	66 men, 49 women	107 men, 32 women	104 men, 43 women	96 men, 55 women
Age	M = 25.09 (SD = 9.1)	M = 32.05 (SD = 14.9)	M = 23.54 (SD = 2.9)	M = 23.46 (SD = 4.8)	M = 24.63 (SD = 7.6)
Level of education completed					
Postgraduate degree	5	1	3	3	5
Undergraduate degree	23	23	45	44	40
Vocational or technical qualification	5	8	11	7	18
Secondary education	44	64	80	91	88
Primary education	1	18	0	1	0
No formal education	0	1	0	1	0
Income					
less than $450	12	36	23	24	11
$451 - $900	31	51	42	50	55
$901 - $1350	18	13	38	36	36
$1351 - $1600	9	5	16	16	27
$1601 - $2250	5	6	8	12	10
More than $2251	3	4	12	9	12
High-Risk Involvement	≥ 39 points = 41 (52.5%)	≥ 4 in GD = 61 (52.9%)	≥ 5 in IGD = 20 (14.3%)	≥ 76 points = 16 (11.2%)	

### MANCOVA

Before conducting the main analysis, assumptions of linearity and multicollinearity among the dependent variables were examined. Correlations ranged from r = –.24 to r = .64, indicating moderate associations within the impulsivity and compulsivity domains, but low correlations between these domains and PRLT performance. This magnitude suggests that the variables share sufficient variance for a multivariate analysis, without excessive redundancy. Box’s test (χ²(112) = 230.19, p < .001) indicated that the covariance matrices differed significantly across groups. To handle this violation of the homogeneity assumption, Pillai’s trace was selected as the multivariate test statistic. Pillai’s trace is widely recognised as the most conservative and robust test against departures from covariance homogeneity, particularly in designs with unequal group sizes, thereby minimising the risk of Type I errors that could arise from the heteroscedasticity.

The multivariate model revealed a significant effect of group on the combined set of dependent variables, V = 0.25, F(28, 2376) = 5.54, p < .001, indicating multivariate differences among the five groups (videogame, gambling, buying, and pornography users) when considering compulsivity, impulsivity, and cognitive flexibility simultaneously. Additionally, there was a significant effect of age, V = 0.03, F(7, 591) = 2.52, p = .015, but not of educational level, V = 0.016, F(7, 591) = 1.4, p = .2.

Follow-up univariate analyses showed significant group effects for all variables except Positive Urgency (F(4, 597) = 2.06, p = .085, η²ₚ = .01). For the case of compulsivity, a large effect size was found (F(4, 597) = 32.93, p < .001, η²ₚ = .18), and small to medium effect sizes were found in Lack of Premeditation (F(4, 597) = 5.69, p < .001, η²ₚ = .04), Lack of Perseverance (F(4, 597) = 4.81, p < .001, η²ₚ = .03), Sensation Seeking (F(4, 597) = 2.67, p = .032, η²ₚ = .02), Negative Urgency (F(4, 597) = 3.79, p = .005, η²ₚ = .02), and overall PRLT performance (F(4, 597) = 3.25, p = .011, η²ₚ = .02). Significant differences were also found in education level for Negative (F(1, 597) = 7.35, p = .007, η²ₚ = .01) and Positive Urgency (F(1, 597) = 3.25, p = .011, η²ₚ = .02), and age for Sensation Seeking (F(1, 597) = 10.21, p = .001, η²ₚ = .02).

### Post Hoc results

Holm-adjusted post hoc comparisons showed that the buying group consistently differed from the other groups across multiple domains. In compulsivity (η²ₚ = .18), buying group scored significantly higher than controls (p < .001, d = 1.51) and gamers (p < .001, d = 0.90), as well as higher than videogame and pornography groups (p < .001, ds ≈ 0.90). Videogame and pornography groups also scored above controls, although with smaller effects (ds ≈ 0.60).

For negative urgency, most pairwise contrasts did not remain significant after correction (see S1 Table from the Supplementary material for the results of all pairwise contrasts). Significant differences were observed between buying and gambling groups (p = .05), with buying showing higher scores (estimate = 0.34, d = 0.43), suggesting moderately greater negative urgency in buying. A slightly stronger effect was found between buying and videogame groups (estimate = 0.43, p < .001, d = 0.55). In contrast, group differences in positive urgency and sensation seeking were negligible.

Several comparisons involving buyers reached significance for lack of premeditation. Only the group of buyers presented a significant difference with the control group, showing higher scores (estimate = −0.35, p < .001, d = 0.54). This group, also showed significantly higher scores than gambling group (estimate = 0.44, p < .001, d = 0.67), videogame (estimate = 0.35, p < .001, d = 0.54), and pornography groups (estimate = 0.38, p < .001, d = 0.58). A similar pattern emerged for lack of perseverance, where buyers again scored significantly higher than controls (estimate = −0.32, p < .001, d = 0.50), gambling (estimate = 0.40, p < .001, d = 0.62), videogame (estimate = 0.30, p = .01, d = 0.46), and pornography groups (estimate = 0.35, p < .001, d = 0.54). The size of these effects was comparable to that seen for lack of premeditation, indicating that buying group also exhibited notably lower perseverance.

Finally, for the global measure of cognitive flexibility (PRLT), significant differences appeared only between buying and the other behavioural groups. Buying group performed worse than videogame group (estimate = −4.77, p = .021, d = 0.45) and pornography users (estimate = −4.43, p = .04, d = 0.42).

### Generalised Linear Mixed-effects Models (GLME)

The full model, which included all main effects and the complete set of two- and three-way interactions among Log-trial, Phase, and group, converged but produced a singular fit due to a near-zero random-intercept variance. Comparison with a model excluding the three-way interaction indicated no loss of fit, Δχ²(12) = 13.82, p = .31, together with a slightly lower AIC for the simpler specification (see Table 2). This model without the three-way interaction was therefore retained as the reference model for evaluating the contribution of the two-way interactions.

**Table 2:** Model Selection for PRLT Performance.

	N Parameters	AIC	BIC	Chisq	Df	P
Saturated model	43	135339	135748			
Model excluding the three-way interaction	31	135329	135624	13.817	12	0.313
Model excluding Phase x Group	19	135329	135510	24.266	12	0.019
Model excluding Log x Group	27	135336	135592	14.882	4	0.005
Model excluding Log x Phase	28	135526	135792	203.27	3	< .001

*Note.* Models excluding two-way interactions were compared to the model excluding the three-way interaction

Each two-way interaction was then examined in turn. Removing the Phase × group interaction significantly reduced model fit, Δχ²(12) = 24.27, p = .019, as did removing the Log-trial × group interaction, Δχ²(4) = 14.88, p = .005. The Log-trial × Phase interaction was essential for capturing trial-related changes across phases; its removal produced a marked deterioration in fit, Δχ²(3) = 203.27, p < .001. Because all three interactions significantly improved the model, their corresponding main effects were retained. The fixed-effects estimates for the final model are presented in Table 3.

**Table 3:** Effect Estimates for Best-Fitting Model of Correct Choices in the PRLT.

	Estimate	SE	Z	p
Intercept	0.148	0.013	11.271	< .001
Log-trial	0.189	0.015	12.495	< .001
C1	−0.119	0.013	−9.018	< .001
C2	0.162	0.013	12.320	< .001
C3	−0.035	0.013	−2.675	0.007
Videogame group	0.041	0.019	2.155	0.031
Gambling group	−0.039	0.020	−1.959	0.050
Buying group	−0.071	0.022	−3.158	0.002
Pornography group	0.045	0.019	2.387	0.017
Log-trial x C1	−0.068	−0.007	10.389	< .001
Log-trial x C2	−0.042	0.007	−6.485	< .001
Log-trial x C3	0.049	0.007	7.476	< .001
Log-trial x videogame	0.020	0.022	0.909	0.363
Log-trial x Gambling	−0.049	0.023	−2.132	0.033
Log-trial x Buying	−0.050	0.026	−1.927	0.054
Log-trial x Pornography	0.014	0.022	0.643	0.520
C1 x videogame	−0.039	0.019	−2.033	0.042
C2 x videogame	0.015	0.019	0.784	0.433
C3 x videogame	0.010	0.019	0.542	0.588
C1 x Gambling	0.037	0.020	1.869	0.062
C2 x Gambling	0.000	0.020	0.025	0.980
C3 x Gambling	0.003	0.020	0.132	0.895
C1 x Buying	0.029	0.022	1.296	0.195
C2 x Buying	−0.001	0.022	−0.028	0.978
C3 x Buying	0.013	0.022	0.590	0.555
C1 x Pornography	−0.035	0.019	−1.877	0.061
C2 x Pornography	0.001	0.019	0.037	0.971
C3 x Pornography	0.010	0.019	0.549	0.583

Given the singularity observed in the original random-effects structure, the final fixed-effects model was refitted with alternative random specifications. A model including only a random slope for Log-trial provided a non-singular solution and yielded identical likelihood and AIC values to the non-singular component of the original model, indicating that the random intercept had contributed no estimable variance. A model including only a random intercept also converged without singularity and provided a lower AIC, but at the cost of discarding meaningful between-participant variability in learning slopes.

## Discussion

Impulsivity, compulsivity, and cognitive flexibility have been considered transdiagnostic variables in addictive disorders [13] and are implicated in both the onset and severity of these disorders [14]. In the last decade, some studies have suggested that excessive and problematic consumption could be considered an addictive behaviour. The present findings provide partial support for the transdiagnostic role of these processes, although the pattern of results suggests important differences across behavioural domains. The aims of the study was to identify differences in compulsivity, impulsivity and cognitive flexibility between participants displaying subclinical addictive behaviours while controlling for age and educational level. For this, a multivariate analysis was conducted to assess whether the five groups (i.e., regular gamblers, video gamers, buyers, porn consumers and a control group) differed. The multivariate test indicated clear overall group differences when all variables were considered jointly, and a smaller but significant contribution of age, whereas educational level was not associated with the combined dependent variables. This overall effect suggests that these psychological domains are not uniformly distributed across behavioural profiles, supporting the notion that different forms of problematic engagement may be characterised by distinct configurations of impulsivity, compulsivity, and cognitive control [43]. Regarding age differences, some studies have shown that there are differences in impulsivity and compulsivity among video game [44] and pornography users among younger people compared to older people [45]. Problematic use of video games and pornography among young adults, and to a lesser extent among gamers, is likely largely due to a generational shift characterised by the emergence of new technologies and free internet access to a wide variety of video games and pornography. Furthermore, several studies have shown that adolescence and early adulthood are periods of heightened vulnerability to the development of mental health disorders [46]. The higher engagement in video games and pornography among younger individuals may also reflect broader generational changes associated with increased access to digital technologies and online content.

Group differences were widespread across the impulsivity–compulsivity dimensions. Compulsivity showed the largest effect, with buyers presenting markedly higher scores than all other groups. This finding suggests that compulsivity may be a particularly salient dimension in differentiating behavioural profiles, especially in comparison to impulsivity-related traits, which showed smaller and more variable effects. This pattern is consistent with previous large-scale findings indicating that compulsivity is more strongly associated with the severity and persistence of behavioural addictions than impulsivity, which may play a more prominent role in earlier or less stable stages of engagement [16,47]. In contrast, smaller group effects emerged for lack of premeditation, lack of perseverance, sensation seeking, negative urgency and global PRLT accuracy, whereas positive urgency did not differ significantly across groups that may indicate that not all components of impulsivity are equally relevant across behavioural domains, reinforcing the importance of conceptualising impulsivity as a multidimensional construct rather than a unitary trait [48]. Additionally, significant associations with education were observed for both urgency facets, and age was related to sensation seeking, consistent with expected developmental influences.

Similarly, post hoc contrasts highlighted a consistent pattern in which buyers displayed elevated compulsivity and higher impulsivity scores in several subdomains. Across these measures, buyers scored higher than controls and also exceeded the other behavioural groups, with effect sizes in the medium to large range. One possible explanation is that compulsive buying involves a reinforcement cycle strongly linked to immediate emotional regulation, where purchasing behaviour serves as a mechanism for mood modification and short-term relief [47]. Differences involving negative urgency were less stable after correction, although buyers tended to show higher scores than gamers and gamblers. For lack of premeditation and lack of perseverance, buyers again showed the most pronounced elevations, exceeding all comparison groups. Finally, in the global measure of cognitive flexibility with the PRLT task, buyers performed more poorly than gamers and pornography users, suggesting a relative disadvantage in adapting to changing reward contingencies even before trial-level learning patterns were examined. These results reinforce the findings of other studies that have reported that compulsive buying presents components of impulsivity and compulsivity similar to addictive disorders [25]. Importantly, when cognitive flexibility was examined at the trial level, group differences in reversal-related processes were minimal, suggesting that the observed differences in overall performance may not reflect fundamental impairments in adaptive learning mechanisms.

In order to evaluate more in depth cognitive flexibility patterns across groups, a GLME model was conducted as described in Jara-Rizzo et al. [36], where trial-by-trial responses are included both as a fixed and random-effect factor. This approach allows for a more fine-grained examination of learning dynamics beyond aggregate performance measures.

Across the task, overall accuracy increased with Log-trial, reflecting a reliable learning effect that was systematically modulated by task structure (Phase). Specifically, the first-versus-second half contrast (C1) revealed that learning improvements were modest initially but became steeper during the latter half of the task (phases 3 and 4) as participants settled into the task demands. Furthermore, as expected, the alternating reversal contrast (C2) demonstrated that the inverted reward structures in phases 2 and 4 not only caused a marked decline in overall accuracy but also significantly slowed the rate of learning. While the final contrast (C3) accounted for additional, non-reversal structural variance, the pattern of accumulated experience during task performance and contingency adaptation remained consistent across all groups. This lack of group-level differences indicates that the fundamental mechanisms underlying probabilistic learning are largely preserved regardless of behavioural profile. Consequently, in subclinical populations, differences in behavioural engagement may not be driven by core impairments in adaptive learning processes, but rather by alterations in other domains such as impulsivity and compulsivity.

Group differences in overall accuracy were small, and the interaction between Log-trial and group suggested that gamblers showed slightly slower improvements than controls. Interactions between Phase and group were generally weak, and none of the reversal-related contrasts differed across groups. This indicates that the cost of adapting to contingency reversals was broadly similar in all five groups, with no evidence that any particular group displayed heightened inflexibility during reversal phases. While existing theoretical models often posit cognitive inflexibility as a core, transdiagnostic feature of addiction, these findings suggest a more nuanced reality. The absence of severe trial-level deficits across groups indicates that cognitive inflexibility may not be a universal prerequisite for the onset of problematic consumption. Instead, these results suggest a need to refine current models, proposing that general cognitive inflexibility might emerge primarily at later stages of clinical severity or manifest selectively in the presence of addiction-specific cues. Consequently, cognitive inflexibility may serve as a context-dependent marker of severity [14,17] rather than an early-stage core trait across all behavioural addictions.

Although the present findings characterise a subclinical population, generalising these cognitive profiles to clinical samples necessitates accounting for the effects of psychotropic medication. Pharmacological treatments, frequently administered alongside psychological interventions for severe behavioural addictions, can significantly modulate dopaminergic and serotonergic pathways, thereby directly altering reward-learning dynamics and either enhancing or blunting cognitive flexibility.

## Conclusions

Excessive consumption has, in some cases, led to problematic behaviours, where individuals exhibit patterns characterised by impulsivity, compulsive tendencies, emotional dysregulation, and difficulty quitting consumption despite negative social and psychological consequences [3]. The present study aimed to identify whether the variables (compulsivity, impulsivity, cognitive flexibility) considered transdiagnostic in addictive disorders are involved in other types of problematic consumption. Specifically, the levels of impulsivity, compulsivity, and cognitive flexibility were compared between 4 groups (regular consumers of video games, gambling, pornography, and purchasing behaviour) and a control group. Intergroup comparison analyses showed that the buyers group exhibited differences across multiple impulsivity domains. For example, negative urgency differed significantly between the buyers’ and gambling groups, with higher scores in the buyers’ group, suggesting a moderately greater negative urgency in this group, and a slightly greater effect between the buyers’ and gambling groups. Regarding comparisons with the control group, significant differences were found only with the buyers’ group, which showed higher scores in negative urgency and lack of premeditation. Higher scores for lack of perseverance were also observed in the buyers’ group compared to all other groups. On the other hand, compulsivity was the trait that showed the greatest impact, with significantly higher scores for buyers than for all other groups. Finally, the results show that the buyers’ group performed worse on the task of measuring global cognitive flexibility compared to the video game and pornography groups. However, no differences were found between the groups during the contingency change phases, indicating that the cost of adapting to contingency changes was generally similar across all five groups.

These findings suggest that impulsivity and compulsivity may manifest differently across these activities, with implications for more tailored intervention strategies that consider both the nature of the disorder and the specific cognitive deficits involved. Regarding the practical implications of the observed effect sizes, the large magnitude of compulsivity in the buying group suggests it constitutes a primary target for early psychological intervention. Conversely, the small effect sizes associated with cognitive flexibility indicate that, at subclinical stages, preventative strategies may not need to prioritise the neurocognitive rehabilitation of reversal learning.

### Limitations of the study

First, it is important to note that while the study targeted a subclinical sample, there was an unequal distribution of participants meeting the criteria for high behavioural severity across the groups. Specifically, both the buying and gambling groups contained a notably higher proportion of participants crossing the problematic threshold compared to the video game and pornography groups. Initially, this imbalance might suggest that the elevated levels of impulsivity, compulsivity, and cognitive inflexibility exhibited by the buyers were simply driven by a higher concentration of pathological cases. However, this severity imbalance alone does not fully explain the results. The gambling group presented a nearly identical proportion of participants with high-risk scores, yet did not exhibit the same extreme cognitive and behavioural profile as the buyers. Consequently, while the overall sample severity remains unbalanced, the distinct neuropsychological alterations observed in the buying group likely reflect core mechanisms unique to that specific behavioural domain, rather than a mere artifact of clinical severity. Furthermore, the distinct diagnostic statuses of these behaviours introduce inherent clinical heterogeneity.

This heterogeneity must be considered when interpreting the findings, as the varied nosological nature of the conditions may partially account for the distinct impulsivity and compulsivity profiles observed across the groups. Also, the reliance on university-based initial recruitment and subsequent snowball sampling introduces inherent selection bias. This sampling strategy yielded a predominantly young and educated cohort, which restricts the representativeness and external validity of the findings. Caution is therefore necessary when generalising these cognitive and behavioural profiles to older, less educated, or broader community populations.

Second, the study relied on cross-sectional data, which precludes the ability to establish causal relationships between the psychological domains evaluated (impulsivity, compulsivity, cognitive flexibility) and the development or persistence of the problematic behaviours. Longitudinal studies employing repeated measures are required to determine whether these cognitive differences represent stable trait markers conferring pre-existing vulnerability, or state-dependent characteristics that emerge as consequences of repetitive behavioural engagement.

Finally, the use of self-report measures introduces the potential for recall bias and social desirability, particularly when assessing sensitive topics such as pornography consumption and gambling. Furthermore, while the online administration of the PRLT allowed for a broader and more diverse sample via snowball sampling, it limited the ability to control for environmental distractions that might have influenced participants’ cognitive performance during the assessment.

## Supporting information

S1 Table Pairwise Contrasts from Post Hoc Analyses. Results from the Pairwise Contrasts from Post Hoc Analyses.
(ODT)

S1 File DF – 4 groups. Data set used in the current study. (CSV)
