# Supplementary material for: Cognitive flexibility, impulsivity, and compulsivity in problematic consumption behaviours
Source: PLoS One. 2026 Jul 23;21(7):e0354596. doi: 10.1371/journal.pone.0354596 (PMC13395419; doi:10.1371/journal.pone.0354596)
Supplement: Supplementary file 1 [file pone.0354596.s001.odt]

Supplementary Materials

Table S1

| variable | contrast | estimate | SE | df | lower.CL | upper.CL | p.value | d |
| --- | --- | --- | --- | --- | --- | --- | --- | --- |
| Compulsivity_GRAC | control - buyers | -23.097 | 2.189 | 597.000 | -29.263 | -16.930 | 0.000 | -1.515 |
| Compulsivity_GRAC | control - gamblers | -9.379 | 2.036 | 597.000 | -15.116 | -3.643 | 0.000 | -0.615 |
| Compulsivity_GRAC | control - gamers | -1.964 | 1.834 | 597.000 | -7.131 | 3.204 | 0.570 | -0.129 |
| Compulsivity_GRAC | control - porno | -9.115 | 1.812 | 597.000 | -14.219 | -4.010 | 0.000 | -0.598 |
| Compulsivity_GRAC | buyers - gamblers | 13.717 | 2.373 | 597.000 | 7.032 | 20.403 | 0.000 | 0.900 |
| Compulsivity_GRAC | buyers - gamers | 21.133 | 2.205 | 597.000 | 14.921 | 27.345 | 0.000 | 1.386 |
| Compulsivity_GRAC | buyers - porno | 13.982 | 2.188 | 597.000 | 7.818 | 20.146 | 0.000 | 0.917 |
| Compulsivity_GRAC | gamblers - gamers | 7.416 | 2.080 | 597.000 | 1.556 | 13.275 | 0.001 | 0.487 |
| Compulsivity_GRAC | gamblers - porno | 0.265 | 2.050 | 597.000 | -5.512 | 6.041 | 0.897 | 0.017 |
| Compulsivity_GRAC | gamers - porno | -7.151 | 1.827 | 597.000 | -12.299 | -2.003 | 0.000 | -0.469 |
| negative urgency | control - buyers | -0.269 | 0.113 | 597.000 | -0.586 | 0.049 | 0.139 | -0.343 |
| negative urgency | control - gamblers | 0.071 | 0.105 | 597.000 | -0.225 | 0.366 | 1.000 | 0.090 |
| negative urgency | control - gamers | 0.162 | 0.094 | 597.000 | -0.104 | 0.428 | 0.495 | 0.207 |
| negative urgency | control - porno | -0.001 | 0.093 | 597.000 | -0.264 | 0.261 | 1.000 | -0.002 |
| negative urgency | buyers - gamblers | 0.339 | 0.122 | 597.000 | -0.005 | 0.683 | 0.051 | 0.433 |
| negative urgency | buyers - gamers | 0.431 | 0.113 | 597.000 | 0.111 | 0.751 | 0.002 | 0.550 |
| negative urgency | buyers - porno | 0.268 | 0.113 | 597.000 | -0.050 | 0.585 | 0.139 | 0.341 |
| negative urgency | gamblers - gamers | 0.092 | 0.107 | 597.000 | -0.210 | 0.393 | 1.000 | 0.117 |
| negative urgency | gamblers - porno | -0.072 | 0.106 | 597.000 | -0.369 | 0.226 | 1.000 | -0.091 |
| negative urgency | gamers - porno | -0.164 | 0.094 | 597.000 | -0.429 | 0.101 | 0.495 | -0.209 |
| positive urgency | control - buyers | 0.050 | 0.089 | 597.000 | -0.200 | 0.299 | 1.000 | 0.080 |
| positive urgency | control - gamblers | 0.024 | 0.082 | 597.000 | -0.208 | 0.256 | 1.000 | 0.039 |
| positive urgency | control - gamers | 0.136 | 0.074 | 597.000 | -0.074 | 0.345 | 0.614 | 0.220 |
| positive urgency | control - porno | -0.070 | 0.073 | 597.000 | -0.277 | 0.137 | 1.000 | -0.113 |
| positive urgency | buyers - gamblers | -0.026 | 0.096 | 597.000 | -0.296 | 0.245 | 1.000 | -0.041 |
| positive urgency | buyers - gamers | 0.086 | 0.089 | 597.000 | -0.165 | 0.338 | 1.000 | 0.140 |
| positive urgency | buyers - porno | -0.120 | 0.089 | 597.000 | -0.369 | 0.130 | 1.000 | -0.194 |
| positive urgency | gamblers - gamers | 0.112 | 0.084 | 597.000 | -0.126 | 0.349 | 1.000 | 0.181 |
| positive urgency | gamblers - porno | -0.094 | 0.083 | 597.000 | -0.328 | 0.140 | 1.000 | -0.152 |
| positive urgency | gamers - porno | -0.206 | 0.074 | 597.000 | -0.414 | 0.003 | 0.056 | -0.333 |
| sensation seeking | control - buyers | 0.241 | 0.110 | 597.000 | -0.069 | 0.550 | 0.259 | 0.315 |
| sensation seeking | control - gamblers | 0.059 | 0.102 | 597.000 | -0.229 | 0.347 | 1.000 | 0.077 |
| sensation seeking | control - gamers | 0.233 | 0.092 | 597.000 | -0.026 | 0.493 | 0.115 | 0.305 |
| sensation seeking | control - porno | 0.036 | 0.091 | 597.000 | -0.220 | 0.292 | 1.000 | 0.047 |
| sensation seeking | buyers - gamblers | -0.181 | 0.119 | 597.000 | -0.517 | 0.154 | 0.639 | -0.237 |
| sensation seeking | buyers - gamers | -0.007 | 0.111 | 597.000 | -0.319 | 0.304 | 1.000 | -0.010 |
| sensation seeking | buyers - porno | -0.205 | 0.110 | 597.000 | -0.514 | 0.104 | 0.436 | -0.268 |
| sensation seeking | gamblers - gamers | 0.174 | 0.104 | 597.000 | -0.120 | 0.468 | 0.574 | 0.228 |
| sensation seeking | gamblers - porno | -0.023 | 0.103 | 597.000 | -0.313 | 0.266 | 1.000 | -0.031 |
| sensation seeking | gamers - porno | -0.198 | 0.092 | 597.000 | -0.456 | 0.061 | 0.259 | -0.258 |
| lack of premeditation | control - buyers | -0.351 | 0.094 | 597.000 | -0.615 | -0.086 | 0.002 | -0.537 |
| lack of premeditation | control - gamblers | 0.089 | 0.087 | 597.000 | -0.157 | 0.335 | 1.000 | 0.136 |
| lack of premeditation | control - gamers | 0.000 | 0.079 | 597.000 | -0.221 | 0.222 | 1.000 | 0.000 |
| lack of premeditation | control - porno | 0.031 | 0.078 | 597.000 | -0.188 | 0.250 | 1.000 | 0.047 |
| lack of premeditation | buyers - gamblers | 0.440 | 0.102 | 597.000 | 0.153 | 0.726 | 0.000 | 0.673 |
| lack of premeditation | buyers - gamers | 0.351 | 0.094 | 597.000 | 0.085 | 0.617 | 0.002 | 0.537 |
| lack of premeditation | buyers - porno | 0.381 | 0.094 | 597.000 | 0.117 | 0.646 | 0.000 | 0.584 |
| lack of premeditation | gamblers - gamers | -0.089 | 0.089 | 597.000 | -0.340 | 0.162 | 1.000 | -0.136 |
| lack of premeditation | gamblers - porno | -0.058 | 0.088 | 597.000 | -0.306 | 0.189 | 1.000 | -0.089 |
| lack of premeditation | gamers - porno | 0.031 | 0.078 | 597.000 | -0.190 | 0.251 | 1.000 | 0.047 |
| lack of perseverance | control - buyers | -0.324 | 0.094 | 597.000 | -0.588 | -0.060 | 0.005 | -0.497 |
| lack of perseverance | control - gamblers | 0.080 | 0.087 | 597.000 | -0.165 | 0.326 | 1.000 | 0.123 |
| lack of perseverance | control - gamers | -0.022 | 0.079 | 597.000 | -0.243 | 0.199 | 1.000 | -0.033 |
| lack of perseverance | control - porno | 0.029 | 0.078 | 597.000 | -0.189 | 0.248 | 1.000 | 0.045 |
| lack of perseverance | buyers - gamblers | 0.405 | 0.102 | 597.000 | 0.119 | 0.691 | 0.001 | 0.620 |
| lack of perseverance | buyers - gamers | 0.302 | 0.094 | 597.000 | 0.036 | 0.568 | 0.010 | 0.463 |
| lack of perseverance | buyers - porno | 0.354 | 0.094 | 597.000 | 0.090 | 0.617 | 0.002 | 0.542 |
| lack of perseverance | gamblers - gamers | -0.102 | 0.089 | 597.000 | -0.353 | 0.148 | 1.000 | -0.157 |
| lack of perseverance | gamblers - porno | -0.051 | 0.088 | 597.000 | -0.298 | 0.196 | 1.000 | -0.078 |
| lack of perseverance | gamers - porno | 0.051 | 0.078 | 597.000 | -0.169 | 0.272 | 1.000 | 0.079 |
| PRLT_GLOBAL | control - buyers | 2.982 | 1.534 | 597.000 | -1.339 | 7.303 | 0.366 | 0.279 |
| PRLT_GLOBAL | control - gamblers | 1.219 | 1.427 | 597.000 | -2.801 | 5.239 | 1.000 | 0.114 |
| PRLT_GLOBAL | control - gamers | -1.793 | 1.285 | 597.000 | -5.414 | 1.829 | 0.818 | -0.168 |
| PRLT_GLOBAL | control - porno | -1.452 | 1.269 | 597.000 | -5.029 | 2.124 | 1.000 | -0.136 |
| PRLT_GLOBAL | buyers - gamblers | -1.763 | 1.663 | 597.000 | -6.447 | 2.922 | 1.000 | -0.165 |
| PRLT_GLOBAL | buyers - gamers | -4.775 | 1.545 | 597.000 | -9.128 | -0.422 | 0.021 | -0.447 |
| PRLT_GLOBAL | buyers - porno | -4.434 | 1.533 | 597.000 | -8.753 | -0.115 | 0.036 | -0.415 |
| PRLT_GLOBAL | gamblers - gamers | -3.012 | 1.457 | 597.000 | -7.118 | 1.094 | 0.314 | -0.282 |
| PRLT_GLOBAL | gamblers - porno | -2.671 | 1.437 | 597.000 | -6.719 | 1.377 | 0.381 | -0.250 |
| PRLT_GLOBAL | gamers - porno | 0.340 | 1.280 | 597.000 | -3.267 | 3.948 | 1.000 | 0.032 |
